# NADPH Oxidase Regulates the Growth and Pathogenicity of *Penicillium expansum*

**DOI:** 10.3389/fpls.2021.696210

**Published:** 2021-08-12

**Authors:** Xuemei Zhang, Yuanyuan Zong, Di Gong, Lirong Yu, Edward Sionov, Yang Bi, Dov Prusky

**Affiliations:** ^1^College of Food Science and Engineering, Gansu Agricultural University, Lanzhou, China; ^2^Department of Postharvest Science of Fresh Produce, Agricultural Research Organization, Volcani Center, Rishon LeZion, Israel

**Keywords:** *Penicillium expansum*, NADPH oxidases, reactive oxygen species, growth, pathogenicity

## Abstract

The occurrence of reactive oxygen species (ROS) during the colonization of necrotrophic pathogens attacking fruit is critical during the attack, but its importance in *Penicillium expansum* remains unclear. This study aimed to determine the regulatory effects of NADPH oxidase (Nox) genes on the growth and pathogenicity of *P. expansum* in apple fruits. Deletion mutants of Δ*PeNoxA*, Δ*PeNoxR*, and Δ*PeRacA* genes were constructed to determine the contribution to the colonization process. The Δ*PeRacA* strain had a significant effect on the reduction of growth and pathogenicity, the Δ*PeNoxA* strain negatively regulated the growth and development of *P. expansum* and did not show any significant effect on the pathogenicity, and the Δ*PeNoxR* strain showed no effect on the growth or pathogenicity of *P. expansum* in the apple fruits. However, analysis of the content of O_2_^–^ and H_2_O_2_ in the mycelium of all the Nox mutants showed a significant reduction, confirming the functionality of Nox mutations. Growth under stress conditions in the presence of Congo red, sodium lauryl sulfate, and H_2_O_2_ showed a negative effect on the radial growth of Δ*PeNoxA*, but a positive effect on radial growth reduction by Δ*PeNoxR* and Δ*PeRacA* mutants was shown. Interestingly, the host antioxidant activity levels of superoxide dismutase (SOD) andcatalase (CAT) in the fruits after inoculation with Δ*PeNoxA*, Δ*PeNoxR*, and Δ*PeRacA* mutants declined, suggesting reduced ROS accumulation in the colonized region. These results suggest that *PeNoxA*, *PeNoxR*, and *PeRacA* differentially regulate the growth and pathogenicity of *P. expansum* by producing ROS, and that *PeRacA* showed the strongest regulatory effect.

## Introduction

*Penicillium expansum* is a necrotrophic pathogen that infects temperate fruits, such as pomes, stones, and berries, through wounds during harvesting and storage (Sun et al., 2018). Evidence of the ability of *P. expansum* to germinate and temporarily grow in a host tissue producing high levels of H_2_O_2_ suggested their role in signaling molecule for the induction of fruit defense (Hadas et al., 2007). Upon initial colonization of a non-host fruit by *P. expansum*, the host releases a large amount of reactive oxygen species (ROS) at the infection site, inhibiting fungal colonization. However, the addition of exogenous catalase (an H_2_O_2_-scavenging enzyme) led to reduced ROS production in the host and enhanced the successful colonization of *P. expansum* on non-host citrus fruits (Macarisin et al., 2007). Studies on the mechanism underlying the sensitivity of *P. Expansum* to intracellular accumulation of *P. expansum* indicate that under high H_2_O_2_-induced oxidative stress, intracellular ROS production by the host is mainly located in the mitochondria. Overexposure of host cells to ROS causes impairments in DNA, lipids, and protein, eventually leading to cell death and progressive aging of an organism (Levine et al., 2000; Xu and Tian, 2008). In order to maintain a stable level of ROS, many organisms have evolved ROS scavenging systems that are mainly enzymatic or non-enzymatic. The enzymatic scavenging system includes superoxide dismutase (SOD) and catalase (CAT), ascorbate peroxidase (APX), etc. SOD causes superoxide conversion into H_2_O_2_, whereas CAT converts H_2_O_2_ into H_2_O. ROS scavenging systems are essential for maintaining ROS levels both in hosts and in pathogens (Wang et al., 2019).

Pathogenic fungi produce ROS by the catalysis of NADPH oxidase (Nox) and play an essential role in their infection processes, signal transduction, and pathogenicity (Tudzynski et al., 2012). In these cases, proper fungal antioxidative systems are expressed in the pathogens to enable fungal development (Lin et al., 2017). Three Nox catalytic subunits, namely NoxA, NoxB, and NoxC, have been found in filamentous fungi, such as *Botrytis cinerea*, *Neurospora crassa*, and *Magnaporthe oryzae* (Kim, 2014). NoxR is a regulatory factor of the Nox catalytic subunit; its coding structure is similar to that of the p67phox protein in mammals (Takemoto et al., 2006). Other Nox regulatory subunits such as Rho3, Cdc42, and RacA also play regulatory roles in fungi (Minz et al., 2013; Si et al., 2016). In the Nox family, only NoxA has a catalytic core domain. The regulatory subunit NoxR and the small GTPases RacA are necessary to activate fungal NoxA function and sometimes act alone (Li et al., 2019).

Studies have reported that NoxA in *Aspergillus nidulans* and *Podospora anserina* had no effect on their asexual development but the affected ROS production and sporophore formation of their hyphae (Lara-Ortíz et al., 2003; Malagnac et al., 2004). In *B. cinerea*, NoxR is responsible for activating the function of NoxA. *NoxR* deletion was shown to severely affect the sexual and asexual development and the sensitivity oxidative stress of *B. cinerea* (Segmüller et al., 2008). The absence of *RacA* in *Epichloë festucae* increased hyphal branching, altered the growth of the hyphal tip, and decreased the ROS content. Furthermore, NoxR could activate NoxA synergistically with RacA (Scott et al., 2007). In *Aspergillus niger*, RacA governs polarity maintenance by controlling actin but not microtubule dynamics, which is consistent with its localization at the hyphal apex. The deletion of *RacA* caused an actin localization defect, leading to the loss in the polarization tip extension of hyphae. Moreover, NoxR is a specific effector of RacA, which plays a critical role in the asexual development of the pathogen (Kwon et al., 2011).

A report has illustrated the effects of Nox on the asexual and sexual development of filamentous fungi (Tudzynski et al., 2012). However, for *P. expansum*, no reports have described the regulatory effects of the Nox family. Using gene knockout and complementation methods, we analyzed the ROS contents and stress resistance of NoxA, NoxR, and RacA on the growth and pathogenicity of *P. expansum*. By studying the production of mutant ROS as well as their growth, morphology and pathogenicity, we determined the effect of *PeNoxA*, *PeNoxR*, and *PeRacA* deletion on the colonization patterns of *P. expansum*.

## Materials and Methods

### Fungal Strain and Growth Condition

*Penicillium expansum* (T01) was obtained from the Institute of Botany, the Chinese Academy of Sciences. A spore suspension was prepared according to Kumar et al. (2017), with minor modifications. Cultures were grown at 25°C in the dark, and maintained on potato dextrose agar (PDA) plates (Beijing Soleibao Technology Co., LTD, China). Then, 10 ml of sterile distilled water supplemented with 0.05% (v/v) Tween 80 (Solarbio, China) by removing the conidia from 7-day-old PDA. A hemocytometer was used to determine the concentrations of the spores. Single-spore cultures were obtained and stored at –80°C until use.

### DNA Extraction

DNA from the WT strain grown in a CY liquid medium (3 g/L NaNO_3_, 1 g/L K_2_HPO_4_⋅3H_2_O, 0.5 g/L KCl, 0.5 g/L MgSO_4_⋅7H_2_O, 0.01 g/L FeSO_4_⋅7H_2_O, 30 g/L sucrose, 5 g/L yeast extract, pH = 5.2) in the dark at 25°C for 48 h and hyphae were collected and stored at –80°C for DNA extraction. According to the instructions of the manufacturer, The DNA was prepared with Fungal DNA Extraction Kit (No. D3195, OMEGA, Guangzhou Feiyang Biological Engineering Co., LTD, China) as described by Yang et al. (2014). The extracted DNA was analyzed by gel electrophoresis and then stored at –20°C until use.

### Gene Knockout and Complementation

**FIGURE 1:**
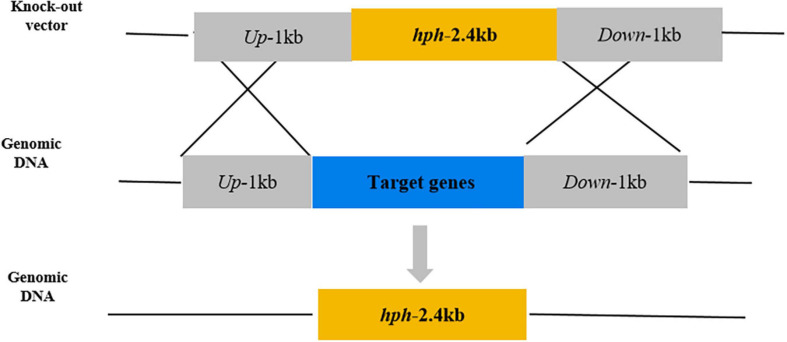
Schematic of the method of gene knockout.

Based on the gene sequences of *PeNoxA* (ID:27678081), *PeNoxR* (ID:27678342), and *PeRacA* (ID:27674691) of *P. expansum* T01 in the NCBI database,^1^ knockout mutants of *PeNoxA*, *PeNoxR*, and *PeRacA* were generated using a homologous recombination strategy and by *Agrobacterium tumefaciens*-mediated transformation, as described by Li et al. (2015). The primers used for the amplification of the up-and downstream sequences are listed in Supplementary Table 1, and the results are shown in Supplementary Figure 1. Gene knockout vectors were constructed by inserting the homologous recombination sequences (approximately 1 k beach), flanking the target genes into the upstream and downstream sides of the hygromycin resistance gene in the vector pCHPH. The homologous recombination knockout strategy is shown in Figure 1. The vectors were transformed into *A. tumefaciens* by freezing-thawing method. During transformation, hygromycin B (Beijing Soleibao Technology Co., LTD, China) was used to select transformants that have undergone resistance screening collection of hyphae and extraction of genomic DNA. Mutants were identified by the difference in the size of amplified fragments between them and the wild type (WT), and the results are shown in Supplementary Figure 2. For the construction of complementation vectors, DNA fragments of *PeNoxA*, *PeNoxR*, and *PeRacA*, namely, the full genomic sequence of the genes and the promoter and terminator regions were cloned into pCNEO-N. G418 (GIBCO, Wolcavi (Beijing) Biological Technology co., LTD, China) was used to select the transformants. The primer sequences used to construct the complementation vectors are shown in Supplementary Table 2.

### Phenotypic Analysis

Colony growth was determined according to the method of Zong et al. (2015), with minor modifications. PDA plates were inoculated with 3 μl of 1 × 10^6^ conidia/ml of either the WT, mutants or complementation strains and incubated at 25°C in the dark. Growth was determined by measuring the diameter after 7 days. Average surface (cm^2^) was used to express colony growth. Each strain had three replicates, and nine plates were used for each replicate.

The rate of spore germination and the length of germ tubes were determined based on the method of Long et al. (2017), with minor modifications. Briefly, 3 μl of each conidial suspension (1 × 10^6^ conidia/ml) was inoculated on PDA plates and incubated at 25°C for 9, 10, and 11 h in the dark. The rate of spore germination and the length of germ tubes of each strain were recorded at different time periods with a microscope (Olympus Corporation, Tokyo, Japan). Each strain had three replicates, and nine plates were used for each replicate.

According to the instructions of the manufacturer, the O_2_^–^ and H_2_O_2_ contents were measured with a kit from Suzhou Comin Biotechnology (Suzhou, China) as described by Zhang et al. (2020). The spore suspension of either the WT, mutants, or complementation strains (3 μl, 1 × 10^6^ conidia/ml) was inoculated on PDA plates, cultured for 24 h, and transferred to a CY medium for 48 h. Then, hyphae were collected to determine the contents of O_2_^–^ and H_2_O_2_. All determination was done at least three times.

Stress resistance was assayed based on Siwy et al. (2016), with minor modifications. Congo red (CR) 25 mg/L, 0.02% (w/v) sodium dodecyl sulfate (SDS), and 100 mM H_2_O_2_ were added to the PDA plates. Each plate was inoculated with 3 μl of each conidial suspension (1 × 10^6^ conidia/ml) and cultured at 25°C in the dark. The morphology of colony growth was recorded, and radial growth was measured after 7 days. Average surface (cm^2^) was used to express colony growth. Each strain had three replicates, and nine plates were used for each replicate.

Spore production was determined according to the method of Zhou et al. (2018), with minor modifications. PDA plates containing 1 × 10^6^ conidia/ml of each strain were incubated at 25°C in the dark for 7 days, and then 5 ml of sterile water (containing 0.05% Tween-20) was added to each plate. Conidia were visualized with a microscope (Olympus Corporation, Tokyo, Japan) and counted with a hemocytometer. Each strain had three replicates, and nine plates were used for each replicate.

### Pathogenicity Experiments

Pathogenicity was determined based on the method of Levin et al. (2019), with minor modifications. The spore suspension (10 μl, 1 × 10^5^ conidia/ml) was inoculated at the wound of the apple fruits (cv. Fuji) using Nichipet EX (Nichiryo, Nagaoka, Japan). The inoculated fruits were packed in polyethylene bags and then stored under ambient conditions (25 ± 2°C, RH 80–85%) in darkness. We evaluated the decayed fruits after 7 days. Colonized area (cm^2^) was used to express wound surface. Each strain had three replicates, and nine fruits were used for each replicate.

According to the instructions of the manufacturer, SOD and CAT activities were measured with the kit from Beijing Solarbio Science and Technology (Solarbio, China) as described by Zhang et al. (2020). The configured spore suspension (10 μl, 1 × 10^5^ conidia/ml) was inoculated at the wound of the apple fruits (cv. Fuji). The inoculated fruits were packed in polyethylene bags and then stored under ambient condition (25 ± 2°C, RH 80–85%) in darkness. Disease-health junction tissues of the apple fruits were collected after 7 days. The SOD activity in the fruits was measured with the SOD kit from Beijing Solarbio Science and Technology. The CAT activity in the fruits was measured with the CAT kit from Beijing Solarbio Science and Technology. All determination was done at least three times.

### Statistical Analysis

All the experiments were repeated at least three times, and the average and standard error (±SE) of the data were calculated using Microsoft Excel 2010. The significance analysis of Duncan’s multiple differences was performed using SPSS 19.0 (SPSS Inc., Chicago, IL, United States) (*P* < 0.05).

## Results

### Effect of *PeNoxA*, *PeNoxR*, and *PeRacA* Knockout on Colony Development and Morphology of the Mutants

**FIGURE 2:**
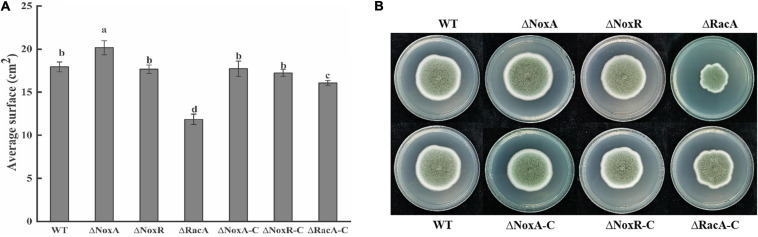
Average surface and morphology of wild type (WT), mutants, and complementation strains. The spore suspension (3 μl) was inoculated on potato dextrose agar (PDA) plates, and cultured at 25°C in the dark. The average surface and morphology were evaluated after 7 days. **(A)** Average surface of WT, mutants, and complementation strains. **(B)** Morphology of WT, mutants, and complementation strains. Bars indicate standard error. Different letters indicate significant differences (*P* < 0.05).

Average surface and colony morphology of Δ*PeNoxR* and their complementation strains indicated that they were similar to those of the WT strain (Figure 2). Δ*PeNoxA* showed a 12% increase compared with the WT, but the surface of complementation strain was similar to that of the WT. However, the average surface of the Δ*PeRacA* mutant was inhibited by 34% with that of WT, but the average surface was regained in the Δ*PeRacA-C* strain (Figure 2A). In terms of colony morphology, the Δ*PeNoxA* and Δ*PeNoxR* mutants did not differ from the WT. The morphology of Δ*PeRacA* showed irregular edges. In terms of appearance, the edges are slightly wrinkled but the morphology was recovered in the Δ*PeRacA-C* (Figure 2B). These results indicated that the *PeNoxA* gene showed a negative regulatory effect on *P. expansum* in surface and colony morphology, while the Δ*PeRacA* strain showed significant inhibition in surface and colony morphology.

### Effect of *PeNoxA*, *PeNoxR*, and *PeRacA* Knockout on Germ Tube Elongation of the Mutants

**FIGURE 3:**
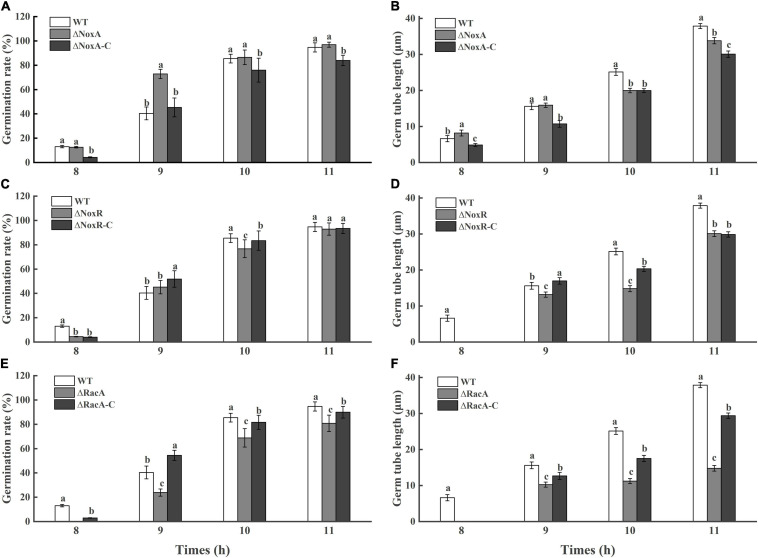
Germination rate and germ tube length of wild type (WT), mutants, and complementation strains. The spore suspension (3 μl) was inoculated on potato dextrose agar (PDA) plates, and cultured for 8, 9, 10, and 11 h at 25°C in the dark. The spore germination and germ tube length were observed under a microscope. Bars indicate standard error. Different letters indicate significant differences (*P* < 0.05). **(A,C,E)**: Germination rate of WT, mutants, and complementation strains. **(B,D,F)**: Germ tube length of WT, mutants, and complementation strains.

The rate of germination in the Δ*PeNoxA* and Δ*PeNoxR* was similar to that in the WT and complementation strains, with minor changes (Figure 3). However, a delay in germination of 20% was observed in the Δ*PeRacA* mutant when compared with the WT and the complementation strain (Figure 3). Also, the length of the germinated tube of the Δ*PeRacA* mutant was inhibited by 61% at 11 h after the initiation of germination, while the complementation strain showed a reduction in inhibition that is not similar to that of the WT strain. The above results also suggested that the spore germination in *P. expansum* was affected in the Δ*PeRacA* mutant.

### O_2_^–^ and H_2_O_2_ Production in the Nox Mutants

**FIGURE 4:**
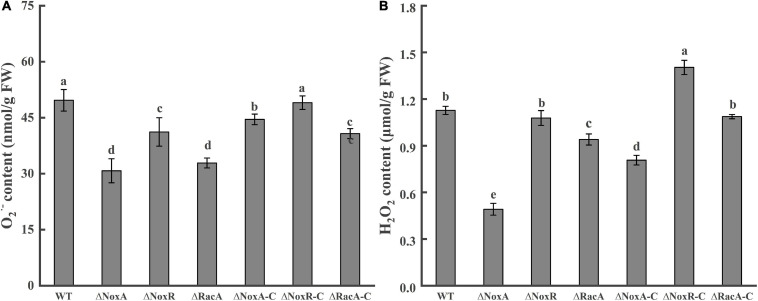
Contents of O_2_^–^ and H_2_O_2_ in the Nox mutants and complementation strains. The spore suspension (3 μl) was inoculated on potato dextrose agar (PDA) plates, cultured for 24 h, and transferred to a CY medium for 48 h. Then, the hyphae were collected to determine the contents of O_2_^–^ and H_2_O_2_. Bars indicate standard error. Different letters indicate significant differences (*P* < 0.05). **(A)** Content of O_2_^–^ of WT, mutants, and complementation strains. **(B)** Content of H_2_O_2_ of WT, mutants, and complementation strains.

Analysis of the O_2_^–^ content in the Δ*PeNoxA*, Δ*PeNoxR*, and Δ*PeRacA* mutants were significantly reduced by 36, 17, and 41%, respectively, when compared with that produced by the WT (Figure 4A). Similarly, the content of H_2_O_2_ in the Δ*PeNoxA* and Δ*PeRacA* mutants were reduced by 56 and 17% compared with that in the WT (Figure 4B). Δ*PeNoxR* was similar to the WT and was recovered in Δ*NoxR-C*. The results indicated that *PeNoxA*, *PeNoxR*, and *PeRacA* genes were involved in the production of ROS in *P. expansum*.

### Oxygen Stress Responses by the Nox Mutants Detected When Grown With Congo Red, Sodium Lauryl Sulfate, and H_2_O_2_

**FIGURE 5:**
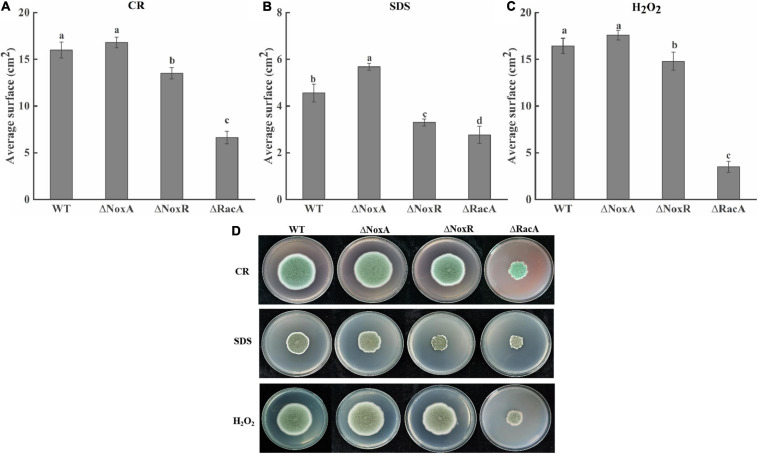
Effect of Congo red (CR), sodium dodecyl sulfate (SDS), and H_2_O_2_ on the colony diameter and colony morphology of wild type (WT) and mutants. CR (25 mg/l), SDS (0.02%), and H_2_O_2_ (100 mM) were added to the potato dextrose agar (PDA) medium. The spore suspension (3 μl) of *P. expansum* was inoculated and cultured at 25°C in the dark for 7 days, and the colony morphology and the average surface was recorded. **(A)** Growth of mutants in the presence of CR. **(B)** Growth of mutants in the presence of SDS. **(C)** Growth of mutants in the presence of H_2_O_2_. Bars indicate standard error. Different letters indicate significant differences (*P* < 0.05). **(D)** The colony morphology of WT and mutants.

The average surface of Δ*PeNoxA* mutants in the presence of SDS was increased by 25% compared with that of the WT, while in the presence of CR and H_2_O_2_ it did not differ from that of the WT. The average surface of the Δ*PeNoxR* and Δ*PeRacA* mutants in the presence of SDS was inhibited by almost 28 and 39%, respectively. Also, the average surface of the Δ*PeRacA* strain in the presence of CR and H_2_O_2_ was inhibited by 58 and 78% compared with the WT (Figure 5). The average surface of the complementation stains reverted, in most cases, the effect of the stresses on their fungal growth (results not shown). The colony morphology of the Δ*PeNoxA* and Δ*PeNoxA-C* strains were not different compared with the WT, while the Δ*PeRacA* mutant was wrinkled with irregular colony edge depressions that become flat in the colony edge of the Δ*PeRacA-C* strain. Δ*PeNoxR* and Δ*PeNoxR-C* showed irregular edges when grown with SDS (Figure 5B). These results indicated that the *PeRacA* gene showed the highest sensitivity to cell wall integrity stress and oxidative stress, followed by the *PeNoxR* and *PeNoxA* genes.

### Effect of *PeNoxA*, *PeNoxR*, and *PeRacA* Knockout on Sporulation and Pathogenicity of the Mutants

**FIGURE 6:**
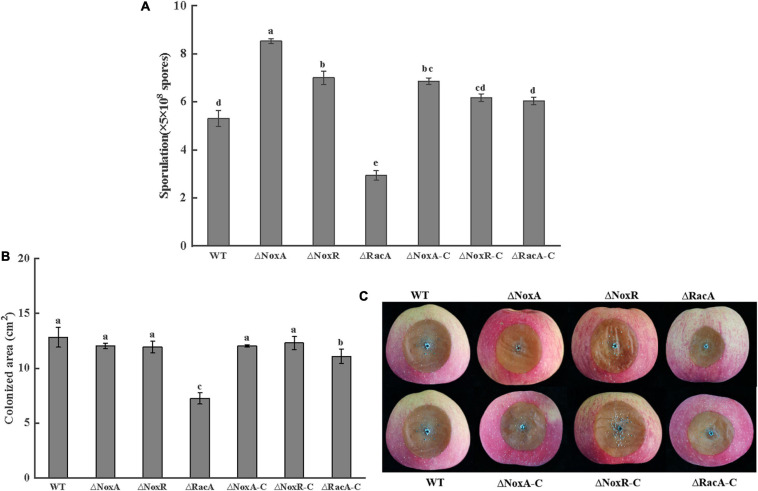
Sporulation and pathogenicity of wild type (WT), mutants, and complementation strains. The spore suspension (3 μl) was inoculated on potato dextrose agar (PDA) plates and cultured for 7 days at 25°C in the dark. The sporulation was recovered by adding sterile distilled water using a bacteriological loop. **(A)** Sporulation of WT, mutants, and complementation strains. **(B)** Colonized area of WT, mutants, and complementation strains. Bars indicate standard error. Different letters indicate significant differences (*P* < 0.05). **(C)** The disease spot form of WT, mutants, and complementation strains.

The level of sporulation of the Δ*PeNoxA* and Δ*PeNoxR* mutants increased by 60 and 30%, respectively, compared with that of the WT, while the sporulation of the Δ*PeRacA* mutant was inhibited by 30%. The sporulation of Δ*PeRacA-C* showed minor differences with the WT strain (Figure 6A). These results indicated that the *PeNoxA* and *PeNoxR* genes showed a negative regulation of the sporulation of *P. expansum*, while the deletion of the *PeRacA* gene enhanced a significant inhibition on sporulation. The colonized area of the Δ*PeNoxA* and Δ*PeNoxR* mutants showed an area similar to that of the WT (Figure 6C), while that of the colonized area of Δ*PeRacA* was inhibited by 43% compared the WT and the Δ*PeRacA-C* strain (Figure 6B). These results indicate that the Δ*PeRacA* mutant is the only one showing a significant contribution to pathogenicity, in comparison with Δ*PeNoxA* and Δ*PeNoxR*.

### Activities of SOD and CAT in Disease-Health Junction Tissues of Apple Fruits of Mutants

**FIGURE 7:**
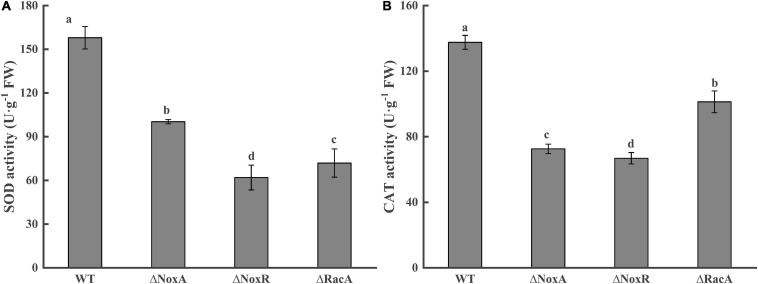
Activities of superoxide dismutase (SOD) and catalase (CAT) at disease-health junction tissues of apple fruits. Bars indicate standard error. Different letters indicate significant differences (*P* < 0.05). **(A)** Activitiy of superoxide dismutase (SOD). **(B)** Activitiy of catalase (CAT).

The colonization of the Nox mutants raised the question if the level of antioxidant activities of the host fruit will be affected by the colonization of the Nox mutant. Evaluation SOD activity responses in the leading edge of the colonized tissue showed that the Δ*PeNoxA*, Δ*PeNoxR*, and Δ*PeRacA* mutants strongly reduced the SOD activity by 36, 60, and 53%, respectively (Figure 7A). A similar pattern was observed when the CAT activity was evaluated at the leading edge of the colonized tissue by the Δ*PeNoxA*, Δ*PeNoxR*, and Δ*PeRacA* mutants showing a reduced CAT activity by 47, 51, and 26%, respectively (Figure 7B). The SOD and CAT activities of the complementation stains reverted in most cases (results not shown). The results indicate that the deletion of the *PeNoxA*, *PeNoxR*, and *PeRacA* genes are modulating the host antioxidant activities as a result of the mutant colonization.

## Discussion

Nox mediates multiple reactions, such as growth of vegetative hyphae, apoptosis, spore fusion, and differentiation of infective structures (Sumimoto, 2008). According to different growth stages, ROS are produced in various intercellular substances, such as peroxidase in vacuoles, and flavin and xanthine oxidase in peroxidase (Susumu et al., 2001; Río et al., 2006). In this study, the colony growth and germination rate of the Δ*PeNoxA* mutant increased when compared with the WT, while there was no significant change in the Δ*PeNoxR* mutant. The colony growth and germination of the Δ*PeRacA* mutant was significantly reduced. Similar results are found in *Epichloë festucae*, *noxA* negatively regulates the asexual development, and the deletion of *noxR* has no effect on growth, while the deletion of *RacA* shows growth defects (Kayano et al., 2013). In *A. nidulans*, NoxA is required for sexual development, RacA activates Nox, and NoxR functions in a parallel pathway that regulates Nox localization (Semighini and Harris, 2008). However, in *B. cinerea* neither NoxA nor NoxB is required for ascospore germination but both are essential for the formation of sclerotia, multicellular sexual structures (Segmüller et al., 2008). These results indicate diverse functions for Nox in different filamentous fungi. Nox catalyzes the ROS production in cells, which can directly act on the cell wall or indirectly act as a second messenger to trigger the hydrolysis and flow of nutrients, thereby weakening nutrients and re-establishing differentiated growth (Malagnac et al., 2004). In *A. nidulans*, NoxA mainly regulates sexual development. However, it would not affect the asexual development of pathogens, which involved ROS production *via* the regulation of mitogen activated protein kinases (MAPK) signals (Eaton et al., 2008). Marschall et al. (2016) reported that Nox activity was strictly regulated involving other membrane proteins, such as protein disulfide isomerase (PDI), specific oxidoreductase (ERO1) and scaffold protein (Iqg1), cytoglobin, and different regulatory domains. Therefore, it is hypothesized that NoxA, NoxR and RacA regulated the growth and development in *P. expansum*. However, whether there is a potential interaction with other regulatory factors requires further research.

Nox is the main enzyme source of ROS in fungi and can use FADH2 and two heme molecules as cofactors. It acts as an electron donor to produce superoxide through electron transfer between membranes. In most cases, oxygen is an electron acceptor, and superoxide is the main product (Rossi et al., 2017). In this study, the contents of O_2_^–^ and H_2_O_2_ in fungi were significantly reduced, indicating that the *NoxA*, *NoxR*, and *RacA* genes affected ROS production in *P. expansum*. Similar results have been reported when deletion of racA resulted in reduced ROS production in *E. festucae* (Tanaka et al., 2008). Segal and Wilson (2018) Nox enzymes produce ROS by transferring electrons from NADPH to molecular oxygen to produce superoxide and other ROS. In *E. festucae*, ROS accumulation was observed in the extracellular matrix of the wild type but not in *noxA* mutants (Tanaka et al., 2006). These observations suggest that the ROS produced by Nox is dispensable for the establishment of fungi growth. In the process of cell differentiation, Nox catalytic subunits respond to internal or external signals, and RacA and NoxR are transferred to the plasma membrane through electrons to form a multi-enzyme complex with complete membrane catalysis, and convert O_2_ to O_2_^–^. The O_2_^–^ can be rapidly converted into H_2_O_2_ quickly by dismutase and diffuse through the membrane as a second messenger and play their roles on fungal cell walls, plasma membrane receptors, or ion channels to activate internal signaling pathways (Scott and Eaton, 2008). Therefore, it is hypothesized that *PeNoxA*, *PeNoxR*, and *PeRacA*, which are primary ROS sources in *P. expansum*, are responsible for intracellular signal transduction and activation of metabolic pathways.

Cell differentiation in fungi has multiple manifestations. It is related to various modes of reproduction and differentiation and various forms of resistance to adverse environmental conditions. Various physiological signals and stresses may cause fungal tissues to undertake specific differentiation processes (Georgiou et al., 2006). NoxA, NoxR, and RacA are the core components of the Nox system; however, there are differences among the Nox mutants, and each of them may have unique functions for different environmental stimuli (Marschall and Tudzynski, 2017). Therefore, to study the effects of the *NoxA*, *NoxR*, and *RacA* genes on various stress responses in *P. expansum*, stress tests were performed. The Δ*PeNoxA* mutant, with the treatment of SDS, CR, and H_2_O_2_, showed an increase in the exogenous stress conditions, whereas the growth of the Δ*PeNoxR* and Δ*PeRacA* mutants showed a reduced stress response. Similar results have been reported for the *noxA* and *noxR* mutants, which displayed cellular sensitivity to H_2_O_2_ and SDS of *A. alternata* (Yang and Chung, 2013). The regulatory functions of NoxA conferring ROS resistance are modulated partially through the activation of the *YAP1-* and *HOG1* MAP kinase-mediated signaling pathways in *A. alternata* (Yang and Chung, 2012). Cellular stress transcription factor *YAP1* plays a global regulatory role in oxidative stress response. Under oxidative stress, *YAP1* enters the nucleus through conformational changes through the formation of disulfide bonds, and activates the expression of Nox (Wood et al., 2003; Lin et al., 2009). The MAPK signaling pathway and Nox complex showed mutual activation effects. The MAPK kinase *Hog1* has also been proven to be resistant to high osmotic pressure and to regulate oxidative stress, and the *Hog1* gene has shown the flexibility and uniqueness of different signaling pathways in response to different stress (Lin and Chung, 2010). Therefore, it is hypothesized that NADPH oxidase may have a regulatory effect on the integrity of the cell wall and oxidative stress response in *P. expansum*, but whether it involves specific interactions with other signaling pathways requires further research.

Reactive oxygen species (ROS) play a significant role during host-pathogen communication. The infection process can be categorized broadly as the recognition phase, host-pathogen communication stage, and the final penetration and infection stage. According to different lifestyles and different infection methods, there are also differences in pathogenicity (Marschall and Tudzynski, 2016). The present results indicate that the pathogenicity of the Δ*PeNoxA* and Δ*PeNoxR* mutants is similar to that of WT, and that the colonization pattern of Δ*PeRacA* is significantly reduced. Similar results were obtained by Chen et al. (2008) during the colonization of rice by *Magnaporthe grisea*. Once the fungal spores are attached to the fruit, they can absorb nutrients from the host for their growth. The chemotropic sensing of nutrients and fungal signals influenced the fungal growth in the fruits (Turrà and Di, 2015). The pathogen counteracts by producing its own ROS to weaken the defense barrier and facilitates the penetration of fruit tissues by specialized infection structures. ROS production plays an important role during infection, and the effects of pathogenicity are often correlated with altered ROS production (Wang et al., 2019). Tanaka et al. (2006) reported that plants infected with the *noxA* mutant lose apical dominance and eventually die in a fungus-perennial *Rye grass mutualistic* interaction. Chen et al. (2008) also observed that *RacA* and PAK kinases *Chm1* and *Nox1* had mutual activation effects. Furthermore, Ygor et al. (2015) found that the pathogenicity of *Colletotrichum gloeosporioides* was directly correlated to H_2_O_2_ production in cowpea. These results demonstrate that fungal ROS production is critical in maintaining a mutualistic fungus-plant interaction. Therefore, we hypothesize that NADPH oxidase may regulate the pathogenicity of *P. expansum*.

Plant cells have an evolved set of defense systems that can effectively eliminate excessive reactive oxygen and maintain stable homeostasis. SOD and CAT are important active oxygen-scavenging enzymes (Chen et al., 2016). We found that SOD and CAT activities decline in fruits during colonization by the Δ*PeNoxA*, Δ*PeNoxR*, and Δ*PeRacA* strains. ROS accumulation is of considerable importance for pathogenic interactions between plants and microorganism (Jones and Dangl, 2006). In the early stages of plant-microorganism interactions, there is a rapid and transient production of ROS (superoxide anion, hydrogen peroxide, and hydroxyl radical) (Buron-Moles et al., 2015). In fact, NOX enzymes often partner with SOD in signaling processes, whereby SOD converts the cell-impermeable superoxide to the diffusible hydrogen peroxide-signaling molecule (Jin et al., 2010). In *Candida albicans*, the deletion of SOD enhanced ROS production during morphogenesis (Rossi et al., 2017). Therefore, it is hypothesized that the Nox of *P. expansum* activates various antioxidant activities in the host.

## Conclusion

Development of the Nox mutants in *P. expansum* showed specific morphological, growth, and colonization responses by *PeNoxA*, *PeNoxR*, and *PeRacA*. The Δ*PeNoxA* mutant negatively regulated the growth and development of *P. expansum*, and showed no effect on pathogenicity. Similarly, the Δ*PeNoxR* mutant showed no significant effect either in pathogenicity or in growth development. On the contrary, the growth development and pathogenicity of Δ*PeRacA* were reduced. Δ*PeRacA* showed the most sensitive to cell wall integrity stress and oxidative stress. Interestingly, the host antioxidant response determined by the analysis of SOD and CAT in fruits showed a reduce response to all the Δ*PeNoxA*, Δ*PeNoxR*, and Δ*PeRacA* mutants, suggesting that ROS play an important role in the interaction between the pathogen and the fruit.

## Data Availability Statement

The original contributions presented in the study are included in the article/Supplementary Material, further inquiries can be directed to the corresponding author/s.

## Author Contributions

XZ and YZ conceived and designed the experiments with the help of DP. YB wrote the manuscript. XZ, DG, and LY performed the experiments. YB, DP, ES, and YZ reviewed and edited the manuscript. All authors contributed to the article and approved the submitted version.

## Conflict of Interest

The authors declare that the research was conducted in the absence of any commercial or financial relationships that could be construed as a potential conflict of interest.

## Publisher’s Note

All claims expressed in this article are solely those of the authors and do not necessarily represent those of their affiliated organizations, or those of the publisher, the editors and the reviewers. Any product that may be evaluated in this article, or claim that may be made by its manufacturer, is not guaranteed or endorsed by the publisher.

## Footnotes

1
https://www.ncbi.nlm.nih.gov/

## Supplementary Material

Supplementary Figure 1 Construction of gene knockout vector.

Supplementary Figure 2 Polymerase chain reaction (PCR) results of gene knockout mutants of NADPH oxidase in *P. expansum*.

Supplementary Table 1 Primers used to amplify upstream and downstream sequences of the target.

Supplementary Table 2 Primer sequences used for complementation vectors.
