# Supplementary material for: NADPH Oxidase Regulates the Growth and Pathogenicity of Penicillium expansum
Source: Front Plant Sci. 2021 Aug 12;12:696210. doi: 10.3389/fpls.2021.696210 (PMC8387719; doi:10.3389/fpls.2021.696210)
Supplement: Supplementary Figure 1 — Construction of gene knockout vector. [file Data_Sheet_1.docx]

Table S1 Primers used to amplify up and down sequences of target genes.

| **Gene** | **Primer sequences (5' - 3')** |
| --- | --- |
| *PeNoxA-up* | F:ACAGCTATGACCATGATTACGAATTCTTTCGTGTCGTGATTGGTGT |
|  | R:GATCCCCGGGTACCGAGCTCGAATTCTAAATGGAACAAGACCTTGG |
| *PeNoxA-down* | F:TTGCCTAACTCGGCGCGCCGAAGCTTGCCAAATCCTCATCAACCAA |
|  | R:GTAAAACGACGGCCAGTGCCAAGCTTGCTGGGAGCTGGCTCTTCTA |
| *PeNoxR-up* | F:ACAGCTATGACCATGATTACGAATTCTCCACGCGATAGACCAGATT |
|  | R:GATCCCCGGGTACCGAGCTCGAATTCGCCTTTGTCGGTACTTTCCA |
| *PeNoxR-down* | F:TTGCCTAACTCGGCGCGCCGAAGCTTAAGAGCGTTCGATGATTTGA |
|  | R:GTAAAACGACGGCCAGTGCCAAGCTTAGCCCATCCTTGTAGTTGTC |
| *PeRacA-up* | F:ACAGCTATGACCATGATTACGAATTCACCCACGTCAAATCACCCTA |
|  | R:GATCCCCGGGTACCGAGCTCGAATTCCAAATCAGATGCCCACCTTC |
| *PeRacA-down* | F:TTGCCTAACTCGGCGCGCCGAAGCTTAGGCAGTCAAATGCAACATC |
|  | R:GTAAAACGACGGCCAGTGCCAAGCTTGCAACCAAGAAAGAAGACCC |

Table S2 Primer sequences used for complementation vectors

| **Gene** | **Primer sequences (5' - 3')** |
| --- | --- |
| *PeNoxA* | NF:GCATGGACGAGCTGTACAAGgagctcatggccgcagctcaagagcc |
|  | NR:ATGGAGCTATTAAATCACTATCTAGACTAAAAGTGTTCCTTCCAGA |
| *PeNoxR* | NF:GCATGGACGAGCTGTACAAGgagctcATGTCGCTCAAACAAGAAAT |
|  | NR:ATGGAGCTATTAAATCACTATCTAGATCAAATCATCGAACGCTCTT |
| *PeRacA* | NF:GCATGGACGAGCTGTACAAGGAGCTCATGGCGACGG GTCCGGCTAC |
|  | NR:ATGGAGCTATTAAATCACTATCTAGACTACAAGATG TTGCATTTGA |


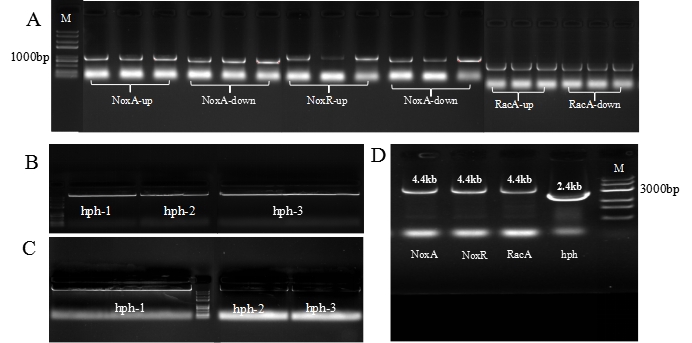


Figure S1 Construction of gene knockout vector. A: The up- and downstream of homologous recombination. B: EcoR I digestion vector. C: Hind III digestion vector. D: PCR identification results.

*PeNoxA-up*, *PeNoxA-down*, *PeNoxR-up*, *NoxR-down*, *PeRacA-up*, *PeRacA-down* gene fragments were cloned from *P. expansum* T01. The vector pCHPH was digested with EcoRI, and the upstream of *PeNoxA-up*, *PeNoxR-up* and *PeRacA-up*were connected to pCHPH, respectively. After the upstream was successfully ligated, the recombinant vector was digested with Hind III, and the downstream of *PeNoxA-down*, *PeNoxR-down* and *PeRacA-down* of the three target genes were respectively connected, and the resulting transformants were identified by PCR**.**


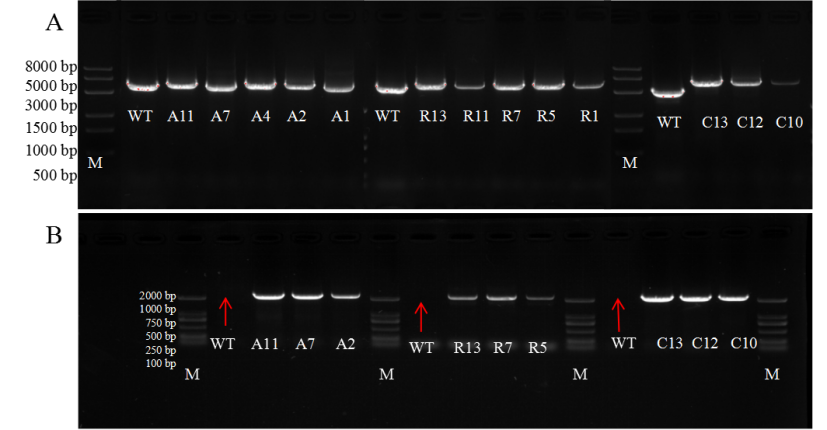


Figure S2 PCR results of gene knockout mutants of NADPH oxidase in *P. expansum*

The positive transformants were identified and screened with three pairs of primers, including *NoxA-up-F* and *NoxA-down-R*, *NoxR-up-F* and *NoxR-down-R*, and *RacA-up-F* and *RacA-down-R*. WT and knockout mutants were amplified by the three primer pairs, and obtain bands of 3.8 kb, 4.4 kb, 3.9 kb, 4.4 kb, 3 kb, and 4.4 kb, respectively. Subsequently, two primer pairs, (*NoxA-up-F* and *TrpC-R*, and *NoxR-up-F* and *TrpC-R*) were used to amplify the strains for verification. No band was found in the WT, while 2.0 kb band was found in the knockout mutants. The mutants obtained by screening were marked as A2, A7, A11, R5, R7, R13, C10, C12, C13.
